# Everyday discrimination and barriers to primary care, mental health, and substance use services: Findings from a community-based cohort of sex workers in Vancouver, Canada (2015–2024)

**DOI:** 10.1371/journal.pgph.0004647

**Published:** 2025-06-16

**Authors:** Kirstin Kielhold, Kate Shannon, Charlie Zhou, Kaylee Ramage, Eileen Pitpitan, Andrea Krüsi, Jennie Pearson, Shira M. Goldenberg

**Affiliations:** 1 San Diego State and University of California San Diego Joint Doctoral Program in Public Health (Global Health), San Diego, California, United States of America; 2 School of Public Health, San Diego State University, San Diego, California, United States of America; 3 Department of Medicine, University of British Columbia, Vancouver, British Columbia, Canada; 4 Department of Public Health, University of Tennessee Knoxville, Knoxville, Tennessee, United States of America; 5 School of Social Work, San Diego State University, San Diego, California, United States of America; 6 School of Criminology, Simon Fraser University, Burnaby, British Columbia, Canada; Aga Khan University, PAKISTAN

## Abstract

We evaluated the association between discrimination and access to primary, mental health, and substance use services among sex workers. Using baseline and semi-annual **questionnaire** data from a community-based cohort of sex workers in Vancouver, Canada (09/2015-02/2024), we used bivariate and multivariable logistic regression with generalized estimating equations to analyze the relationship between discrimination and access to primary care, mental health, and substance use services. Among 518 participants (2768 observations), the median discrimination score was 19 (IQR:11–25), indicating substantial discrimination. In separate multivariate models, every one-point increase in discrimination was associated with increased odds of experiencing barriers to health services (adjusted odds ratio (AOR):1.03, 95%CI:1.02-1.04), unable to access health services when needed (AOR:1.03, 95%CI:1.01-1.04), unmet need for mental health services (AOR:1.04, 95%CI:1.03-1.06), experiencing barriers to counseling for sexual trauma (AOR:1.04, 95%CI:1.02-1.05), and unmet need for substance use treatment (AOR:1.07, 95%CI:1.04-1.09). Discrimination is highly prevalent and associated with reduced access to primary, mental health, and substance use services among sex workers. There is a need for anti-discrimination efforts, including provider training and sex worker partnerships in primary care, alongside policy reforms.

## Introduction

Globally, sex workers face a disproportionate burden of health inequities due to intersecting structural barriers including discrimination and criminalization of sex work [1,2]. In Canada and elsewhere, criminalization has been associated with severe gaps in sex workers’ ability to report violence to the police [3]. Sex work criminalization is influenced by structural and societal contexts of sex workers being highly stigmatized, which is rooted in narratives and stereotypes positioning sex workers as victims [4]. While stigma and discrimination are interwoven concepts, discrimination represents enacted stigma [5]. Discrimination has been measured in many different ways—both experienced and perceived—and can be conceptualized as a social process that results from interpersonal inequalities experienced in people’s lives [6]. Discrimination has been shown to be inversely related to health [7] across different communities and geographical regions [8–10], and across different populations, including people of color [8] and people who inject drugs [10]. Among people who inject drugs, discrimination based on drug use has been associated with increased odds of overdosing, abscesses or infections from injecting, and adverse mental health [10]. However, limited quantitative research has addressed discrimination or its health consequences among sex workers, with a particular dearth of studies using validated measures [9].

Current evidence from the United States has shown that discrimination has been identified as a risk factor for poor health markers and outcomes including chronic self-rated health [11], cardiovascular disease [12], higher use of emergency departments (a measure of poor health access), chronic disease such as high cholesterol, diabetes, heart disease, stroke, and asthma [13], birth defects, and mental health diagnosis [8,14]. Discrimination research to date has been studied with varying measures and mostly in cross-sectional analyses [10,13,14], leaving a need for prospective longitudinal evidence utilizing validated discrimination measures.

Access to care is an important predictor of health which may be adversely affected by discrimination, particularly when experienced within healthcare settings. Experiencing discrimination or the fear of discrimination within healthcare institutions can act as a barrier to care for marginalized populations including women [15], immigrants [15] and people experiencing mental illnesses [16]. Barriers to care may be exacerbated due to the fear of further discrimination and mistreatment after previous exposure to discrimination from healthcare providers [17,18]. Prior qualitative research, mostly focused on HIV, suggests that avoidance of services due to discrimination poses a barrier to care for sex workers in low and middle-income countries [9], including Pakistan [19], India [20], and Nigeria [21]. A systematic review of sex workers’ uptake of mental healthcare services among 32 studies found discrimination, stigma, and mistrust of mental health service providers to all be barriers to care [22]. However, existing research exploring sex worker discrimination is mostly qualitative and largely focuses on outcomes of HIV services, the impact of perceived or real fear of discrimination, or is conducted in lower income countries [19–21]. There is limited quantitative data on the impact of lived experiences of everyday discrimination on sex workers’ health service access, especially in high income countries. Given the burden of physical, mental health, and substance use-related challenges faced by sex workers, there is a clear need for prospective epidemiologic evidence, that extends beyond HIV services, in high-income countries.

As the health inequities which sex workers face are known to be strongly shaped by structural factors such as criminalization, unsafe work environments, and stigma [1]—as opposed to inherent to sex work or individual behaviors—this study draws on a structural determinants of health framework [23], which considers macrostructural, community level, and work environment-level factors, as well as Anderson’s health service utilization model [24]. In Canada, ‘end-demand’ legislation currently criminalizes purchasing sex and various third party activities (i.e., security and other personnel at sex-selling establishments) related to facilitation of the purchasing of sex, while leaving the sale of sex legal under certain circumstances [1,25]. Punitive laws and policies have been identified as key ‘upstream’ macrostructural determinants that shape stigmatizing and discriminatory attitudes towards sex work. As ‘end-demand’ models gain popularity among policy-makers globally, an evaluation of the association between sex work everyday discrimination and sex workers’ health access in ‘end-demand’ settings is needed [26,27]. Previous research in Canada has shown that these laws have resulted in ongoing harms for sex workers. For example, after the instatement of ‘end-demand’ laws, 26.4% of sex workers in Vancouver reported that the new approach to regulating sex work had led to negative changes in their working conditions [3].

Given the burden of discrimination sex workers face and the lack of quantitative research to date on sex workers’ experienced discrimination, using validated measures of discrimination, we evaluated the longitudinal association between discrimination and access to primary, mental health, and substance use services among a cohort of sex workers in Vancouver, Canada over an 8.5-year period.

## Methods

### Study design

AESHA is an open community-based cohort that initiated recruitment in late January 2010. Detailed methods are described elsewhere [28]. Briefly, baseline eligibility criteria included: self-identifying as a woman (both cisgender and transgender women); having exchanged sex for money within the last 30 days; 14 + years old; and, able to provide written informed consent. Given the challenges of recruiting sex workers in isolated and hidden locations, time-location sampling was used to recruit women sex workers through day and late-night outreach to outdoor/public sex work locations (e.g., streets, alleys) and indoor sex work venues (e.g., massage parlors, micro-brothels, and in-call locations) across Metro Vancouver. Online recruitment was used to reach sex workers working through online solicitation spaces. Indoor sex work venues and outdoor solicitation spaces (‘strolls’) were identified through outreach mapping conducted together with current/former sex workers and were updated by the outreach team. This study holds ethical approval through the Providence Health Care/University of British Columbia (UBC) Research Ethics Board (REB number H09-02803).

### Data collection

Following informed written consent, participants completed interview-administered questionnaires at baseline and semi-annually. Interviews were conducted at study offices in Metro Vancouver or a confidential space of the participants’ choice (e.g., home, work). The questionnaire was administered via UBC’s secure RedCap platform by trained interviewers (both sex workers and non-sex workers) and participants received voluntary HIV/STI/HCV serology testing by a trained sexual health research nurse. Measures included socio-demographics, sex work patterns, drug use patterns, physical work environment factors, management structure, access to condoms and other prevention resources, social factors, occupational violence, and utilization of primary care, mental health, substance use, and HIV, STI and Hepatitis C services. At inception (January 2010), participants received a $40 honorarium for each baseline or semi-annual follow-up visit. From September 2021 to March 2022, this amount was adjusted to $65 per visit, given the increased burden to participants of revised questionnaires which were expanded to address impacts of the COVID-19 pandemic. In May 2022, this was further increased to $80 per baseline visit and $65 per semi-annual follow up visit, to account for inflation and participant burden related to questionnaire length.

### Measures

All study measures were assessed at baseline and semi-annually as time-updated variables using a six-month recall period, except for time-fixed demographics (e.g., race). All variables were coded as binary measures (yes vs. no), with the exceptions of everyday discrimination and age, which were analyzed as continuous variables, as well as racialization and primary place of soliciting clients, which were analyzed as 3-way categorical variables.

#### Dependent variable.

We assessed five separate health services outcome variables: 1) *Experienced any barriers to receiving health care* (defined as ‘yes’ to any option including barriers relating to ‘Approachability’, ‘Acceptability’, ‘Availability and accommodation’, ‘Affordability’, ‘Appropriateness’, and ‘Stigma and discrimination’ when asked, ‘what barriers to receiving healthcare have you experienced? (e.g., primary care, hospital, medical care)’), 2) *Unable to access to health services when needed* (defined as ‘Sometimes (25-75% of the time)’, ‘Occasionally (under 25% of the time)’, or ‘Never’, when asked ‘How often can you get healthcare services when you need it?’), 3) *Unmet need for mental health services* (defined as ‘yes’ to unable to access mental health services, including any of: ‘Mental health medications’, ‘Mental health assessment/diagnosis’, ‘Mental health counseling or other support’, ‘Elders or Knowledge Keepers (Indigenous participants only)’, ‘Other Indigenous-led service (Indigenous participants only)’), 4) *Experienced barriers to counseling for sexual trauma* (defined as ‘yes’ to either ‘have you experienced any barriers to counseling or therapy for sexual abuse?’ or ‘have you experienced any barriers to counseling or therapy for these [trauma of violence you may have experienced] experiences?’ ‘Experienced barriers to counseling or therapy for sexual abuse/ violence/ trauma’), and 5) *Unmet need for substance use treatment* (defined as ‘yes’ to ‘In the last 6 months, have you ever tried to access drug or alcohol treatment and been unable to?’).

#### Independent variable.

Discrimination was measured using the Everyday Discrimination Scale^1^ adapted from Williams and colleagues (1997) (α = 0.88) [29]. We used the scale as a continuous measure, with participants receiving a score between 9 and 45, with 9 indicating no reported discrimination and increasing scores with higher levels of discrimination. The scale (S1 Table) was pilot tested, adapted, and validated with our study population (α = 0.93) and was added to the questionnaires in September 2015. Sample response options included, ‘People call you names’, ‘People threaten or harass you’, and ‘People act like they are better than you’. For sex workers who reported ‘sometimes’, ‘usually’, or ‘ever’ to any of the response options, they were asked ‘What do you think is the main reason for those experiences?’ Options were: ‘ancestry or national origins (ethnicity)’, ‘race’, ‘gender identity’, ‘sexual identity’, ‘age’. ‘religion’, ‘physical appearance’, ‘education or income level’, ‘sex work’, ‘drug use’, ‘HIV status’, ‘Environment/other people’s problems’, or ‘other’, more than one option was selected if participant volunteered.

#### Potential confounders.

Drawing on a structural determinants framework, potential confounders were identified via Directed Acyclic Diagrams (DAGs) and review of the literature [30]. Potential confounders are described in further detail in Table 1.

**Table 1:** Potential confounder definitions.

Individual Confounders	Definitions ‘Yes’ defined as
Age	Time updated at follow-up based on age at baseline and interview date
Non-injection drug use^*^ (excluding cannabis and alcohol)	‘Crack cocaine’, ‘Cocaine (powder, snorted, etc.)’, ‘Crystal Meth (side)’, ‘Street methadone’, ‘Heroin’, ‘Benzos/Valium/ Ativan’, ‘Ritalin/Dexedrine’, ‘Percs/ Vicodin/ Demerol’, ‘Dilaudid’, Morphine’, ‘Oxycontin’, ‘T3s and T4s’ ‘Ecstasy’, ‘Special K’, ‘GHB’, ‘relevant prescription drugs’ ‘Inhalants (e.g., poppers)’, ‘Hallucinogens’, ‘Fentanyl’, or relevant ‘Other’ to ‘which non-injection drugs have you used?’
Injection-drug use^*^	‘Heroin alone’, ‘Cocaine alone’, ‘Speedballs’, ‘Street methadone’, ‘Morphine’, ‘Cyrstal meth’, ‘Goofballs’, ‘Dilaudid’, ‘Crack’, relevant ‘Prescription drugs’, ‘Fentanyl’, or relevant ‘Other’ to ‘which injection drugs have you used?’
**Structural Factors**	
Racialization	
*White*	‘White’
*Indigenous*	‘First Nations’, ‘Metis’, or ‘Inuit’
*Other Woman of color* ^2^	‘Asian’, ‘Latinx’, ‘Black’, or ‘Other’
Im/migrated to Canada	‘No’ to the question ‘Born in Canada’
Sexual minority	‘Gay’, ‘Lesbian’, ‘Bisexual’, ‘Asexual’, ‘Queer’, ‘Two-Spirit’, or ‘Other’ vs. ‘straight’ at any study visit
Gender minority	‘Transgender’, ‘Intersex’, ‘Transexual’, ‘Genderqueer’, ‘Two-Spirit’, ‘Trans’, ‘Non-binary’, ‘Demigirl’, or ‘Other’ vs. ‘Cisgender’ at any study visit
Diagnosed with any mental health issue ever	Any one or more of the following possible responses: ‘Depression, Anxiety, Post-traumatic stress disorder, Schizophrenia/schizoaffective, Bipolar disorder, Psychosis (drug-induced), other’, to the question ‘Ever diagnosed with this condition’
Unstable housing^*^	‘Sleeping overnight at a single room occupancy hotel’, ‘Supportive housing’, ‘Modular housing’, ‘With parents’, or ‘With family/relatives’
Reporting feeling in danger where you sleep^*^	Reporting ‘Always’, ‘Usually’, ‘Sometimes’, or ‘Occasionally’
Experienced violence from aggressor posing as client^*^	‘Abducted/kidnapped,’ ‘Raped,’ ‘Strangled,’ ‘Physically assaulted/beaten,’ ‘Locked/trapped in a car,’ ‘Assaulted with weapon,’ ‘Drugged,’ ‘Trapped in room/hotel/housing etc.,’ or relevant ‘Other’ to the question, ‘Have you experienced any of the following bad dates/violence by clients?’
Male intimate partner violence^*^	‘Yes’ to any options under ‘Moderate Physical IPV’, ‘Severe Physical IPV’, ‘Sexual IPV’, ‘Emotional IPV’ and ‘Yes’ to ‘Had any intimate cis man partner, including casual partners’ in the last 6 months
Any violence by any perpetrator^*^	‘Yes’ to any options of ‘Male intimate partner violence’, ‘Sexually assaulted by anyone other than an intimate male partner or aggressor posing as a client’, or ‘Experinced violence from aggressor posing as client’
**Workplace Factors**	
Primary place of soliciting clients^*^	
*Street/Public*	‘Street/outdoor public space’
*Indoor (Informal or Formal)*	‘Crack/drug house’, ‘Bar/night club’, ‘Exotic dance/strip club/ show lounge’, ‘Massage/beauty parlor’, ‘Micro-brothel’, ‘Managed indoor space’, ‘SRO/supportive housing’, or ‘Regular(s) stopped by (home/apartment)’
*Independent*	‘Escort agency’, ‘Newspaper ads’, ‘Online’, ‘1-800 phone chat service’, ‘Personal phone/texting’, ‘Arranged by manager/pimp’, or ‘Arranged by friend’

* Time updated to last 6 months

#### Statistical analysis.

Analyses were restricted to the period during which the everyday discrimination scale was administered (September 1, 2015-February 29, 2024). Baseline descriptive statistics were calculated for all variables. Additionally, frequencies and proportions were calculated for categorical variables and measures of central tendencies (i.e., median, interquartile range (IQR)) were calculated for continuous variables. The primary exposure and potential confounding variables were stratified by each of the five individual outcomes of interest and compared using Pearson’s chi-square test for categorical variables and the Wilcoxon rank-sum test for continuous variables. Bivariate analyses used logistic regressions with generalized estimating equations (GEE) and an exchangeable correlation matrix to account for repeated measurements amongst participants over time. Bivariate analyses examined the association of everyday discrimination and each of the five access outcomes individually. A multivariable confounder model was subsequently built, which included hypothesized individual and structural confounders. We chose to leave out confounders of race, non-injection drug use, sexual minority, and gender minority as these are identities people often face discrimination for. We did not want to obscure the effects of these factors and did not include them in our final model to avoid over adjusting. Models were restricted to those who answered the Everyday Discrimination Scale at least once and reported recent sex work (defined as ‘exchanged sex for money, goods, or services’) (N = 518). Additionally, analyses examining the outcome of ‘experienced any barriers to counseling or therapy for sexual abuse/violence/trauma’ were further restricted to those who reported any lifetime violence (N=460) and analyses examining the outcome of “unmet need for substance use treatment’ were further restricted to those reported recent drug use drug use (N = 403). Statistical analyses were performed in SAS version 9.4 (SAS, Cary, NC), and all p-values are two-sided.

## Results

At enrollment, median everyday discrimination score among 518 sex workers was 19 (11–25 IQR). (Table 2) Almost half, 46.0% identified as Indigenous, 32.4% White, 19.7% as Asian, Latinx, or Other, and 1.7% as Black. For primary place of solicitation, 34.4% reported street/public, 20.9% reported indoor (informal or formal), and 41.7% reported independent. At enrollment, 167 sex workers reported no barriers to receiving healthcare and 424 reported having access to healthcare when needed. Among participants who reported any discrimination (n = 412), the most common self-reported reasons for discrimination at any visit included environment/other people’s problems^3^ (71.4%, n = 370), drug use (38.4%, n = 199), sex work (31.7%, n = 164), physical appearance (41.7%, n = 216), race (23.0%, n = 119), education or income level (20.5%, n = 106), gender identity (20.3%, n = 105), and ancestry/national origins (ethnicity) (20.3%, n = 105). In sub-analysis, among 376 participants who were asked additional questions on perpetrators of discrimination (asked between 2020–2024), 35.6% reported experiencing discrimination by police and 54.8% by healthcare providers during this timeframe.

**Table 2:** Baseline characteristics of women sex workers in Vancouver, Canada, stratified by outcomes of barriers to and unmet need for primary care and mental health service outcomes*, AESHA, Vancouver, Canada (2015-2024).

Characteristic	Total (%)	Experienced any barriers to receiving healthcare^*^		Unable to access health services when needed^*^		Unable to access mental health services^*^	
	(*N *= 518)	Yes (*%*)	No (%)	Yes (*%*)	No (%)	Yes (*%*)	No (%)
		(*n* = 348)	(*n* = 167)	(*n* = 91)	(*n* = 424)	(*n* = 126)	(*n* = 377)
**Primary Exposure of Interest**							
Everyday discrimination^*^ (med, IQR)	19 (11-25)	21 (12-27)	15 (9-23)	22 (15-27)	19 (10-25)	25 (19-29)	17 (9-24)
**Individual Factors**							
Age (med, IQR)	40 (33-47)	40 (32-47)	41 (34-48)	41 (34-48)	40 (32-47)	36 (31-44)	41 (34-48)
Non-injection drug use^*^	307 (59.3)	208 (59.8)	97 (58.1)	54 (59.3)	252 (59.4)	91 (72.2)	207 (54.9)
Injection drug use^*^	238 (46.0)	170 (48.9)	68 (40.7)	42 (46.2)	195 (46.0)	72 (57.1)	158 (41.9)
**Structural Factors**							
Race							
*White*	168 (32.4)	112 (32.2)	55 (32.9)	23 (25.3)	145 (34.2)	48 (38.1)	112 (29.7)
*Indigenous*	238 (46.0)	158 (45.4)	79 (47.3)	47 (51.7)	190 (44.8)	68 (54.0)	167 (44.3)
*Black, Asian, Latinx, Other*	111 (21.4)	77 (22.1)	33 (19.8)	21 (23.1)	88 (20.8)	10 (7.9)	98 (26.0)
Im/migrated to Canada	113 (21.8)	78 (22.4)	34 (20.4)	21 (23.1)	90 (21.2)	12 (9.5)	98 (26.0)
Sexual minority	266 (51.4)	182 (52.3)	82 (49.1)	50 (55.0)	215 (50.7)	71 (56.4)	187 (49.6)
Gender minority	77 (14.9)	55 (15.8)	22 (13.2)	15 (16.5)	61 (14.4)	23 (18.3)	50 (13.3)
Diagnosed with any mental health issue	313 (60.4)	227 (65.2)	85 (50.9)	61 (67.0)	251 (59.2)	98 (77.8)	210 (55.7)
Unstable housing^*^	417 (80.5)	290 (83.3)	125 (74.9)	70 (76.9)	346 (81.6)	114 (90.5)	293 (77.7)
Experienced violence from aggressor posing as client^*^	44 (8.5)	36 (10.3)	7 (4.2)	15 (16.5)	29 (6.8)	16 (12.7)	25 (6.6)
Any male intimate partner violence^*^	41 (7.9)	33 (9.5)	8 (4.8)	10 (11.0)	31 (7.3)	13 (10.3)	27 (7.2)
Any violence by any perpetrator^*^	126 (24.3)	97 (27.9)	28 (16.8)	31 (34.1)	95 (22.4)	50 (39.7)	71 (18.8)
Primary place of solicitation^*^							
*Street/public*	178 (34.4)	120 (34.5)	58 (34.7)	32 (35.2)	146 (34.4)	51 (40.5)	123 (32.6)
*Indoor (informal or formal)*	108 (20.9)	75 (21.6)	31 (18.6)	20 (22.0)	86 (20.3)	20 (15.9)	84 (22.3)
*Independent*	216 (41.7)	145 (41.7)	70 (41.9)	38 (41.8)	177 (41.8)	51 (40.5)	158 (41.9)

* Time updated to last 6 months

NOTES: Non-injection drug use excludes alcohol and cannabis

All variables binary unless otherwise indicated

Over the 8.5-year study period, 86.9% of participants reported experiencing any barriers to healthcare, 39.2% were unable to access health services when needed, 52.9% faced unmet need for mental health services, 47.2% of those experienced barriers to counseling for sexual trauma, and 24.6% of those using drugs faced unmet need for substance use treatment. The range of affirmative responses to the discrimination scale subcategories at least once was 63.4-77.2%, with the most frequently reported being “people act like they are better than you” (77.2%), “people do not treat you with respect (76.4%), and “people are not polite to you” (76.4%) (S1 Table).

At baseline, sex workers experiencing poor access to and unmet need for primary, mental health, and substance use services were more likely to be diagnosed with a mental health issue, use non-injection or injection drugs, and to have experienced violence across occupational, intimate, and community contexts, compared to those who did not experience barriers to or unmet need for primary, mental health, and substance use services (Table 2 and Table 3).

**Table 3:** Baseline characteristics of women sex workers in Vancouver, Canada, stratified by outcomes of access to mental health and substance use services, AESHA, Vancouver, Canada (2015-2024).

	Experienced any barriers to counseling or therapy for sexual abuse/violence/trauma*1			Unmet need for substance use treatment*2		
	Total (%) (N = 460)	Yes (*%*) (n = 75)	No (%) (n = 373)	Total (%) (*N *= 403)	Yes (*%*) (n = 40)	No (%) (n = 354)
**Primary Exposure of Interest**						
Everyday discrimination^*^ (med, IQR)	20 (13-26)	21(15-28)	20 (13-25)	21(15-26)	29 (23-34)	21 (14-25)
**Individual Factors**						
Age (med, IQR)	40 (32-47)	38 (31-43)	40 (32-47)	39(32-47)	38 (29-44)	39 (32-47)
Non-injection drug use^*^	302 (65.7)	54 (72.0)	239 (64.1)	342 (84.9)	35 (87.5)	298 (84.2)
Injection drug use^*^	236 (51.3)	40 (53.3)	190 (50.9)	248 (61.5)	28 (70.0)	213 (60.2)
**Structural Factors**						
Race						
*White*	167 (36.3)	28 (37.3)	134 (35.9)	161 (40.0)	11 (27.5)	148 (41.8)
*Indigenous*	230 (50.0)	45 (60.0)	182 (48.8)	221 (54.8)	28 (70.0)	187 (52.8)
*Black, Asian, Latinx, Other*	62 (13.5)	NS	56 (15.0)	20 (5.0)	NS	19 (5.4)
Im/migrated to Canada	64 (13.9)	NS	58 (15.6)	24 (6.0)	NS	22 (6.2)
Sexual minority	251 (54.6)	48 (64.0)	197 (52.8)	223 (55.3)	24 (60.0)	194 (54.8)
Gender minority	75 (16.3)	16 (21.3)	58 (15.6)	60 (14.9)	NS	51 (14.4)
Diagnosed with any mental health issue	303 (65.9)	60 (80.0)	238 (63.8)	281 (69.7)	34 (85.0)	242 (68.4)
Unstable housing^*^	394 (85.7)	67 (89.3)	317 (85.0)	365 (90.6)	39 (97.5)	318 (89.8)
Experienced violence from an aggressor posing as client^*^	50 (10.9)	12 (16.0)	34 (9.1)	39 (9.7)	9 (22.5)	30 (8.5)
Any male intimate partner violence^*^	43 (9.4)	9 (12.0)	32 (8.6)	40 (9.9)	8 (20.0)	30 (8.5)
Any violence by any perpetrator^*^	132 (28.7)	28 (37.3)	99 (26.5)	121 (30.0)	20 (50.0)	98 (27.7)
Primary place of soliciting clients^*^						
*Street/public*	176 (38.3)	30 (40.0)	141 (37.8)	174 (43.2)	27 (67.5)	143 (40.4)
*Indoor (informal or formal)*	65 (14.1)	10 (13.3)	54 (14.5)	31 (7.7)	NS	29 (8.2)
*Independent*	204 (44.4)	32 (42.7)	167 (44.8)	185 (45.9)	10 (25.0)	172 (48.6)

* Time updated to last 6 months

1 N = 460, 2N = 403

NOTE: Non-injection drug use excludes alcohol and cannabis

NS (Number Suppressed): cell sizes with response options with n < 5 have been suppressed for participant confidentiality

In bivariate GEE analysis, everyday discrimination was associated with experiencing poor access to and utilization of primary, mental health, and substance use services, as were injection drug use and experiencing violence from an aggressor posing as a client. Identifying as a sexual minority was associated with experiencing poor access to primary and mental health services and unstable housing was associated with an unmet need for substance use services. Both intimate male partner violence and violence from any perpetrator were associated with all five outcomes of poor access to services. (Table 4).

**Table 4:** Bivariate GEE analysis of the association between everyday discrimination and barriers to accessing primary, mental, and substance use services among women sex workers in Vancouver, Canada, AESHA, 2015-2024.

	Odds Ratio (95% Confidence Interval)				
Characteristic	Experienced any barriers to receiving healthcare*1	Unable to access health services when needed*1	Unable to access mental health services1	Experienced any barriers to counseling or therapy for sexual violence*2	Unmet need for substance use treatment*3
**Primary Exposure of Interest**					
Everyday discrimination*	1.03 (1.02-1.04)	1.03 (1.01-1.04)	1.04 (1.03-1.06)	1.04 (1.03-1.05)	1.07 (1.05-1.09)
**Individual Factors**					
Age	0.96 (0.95-0.97)	1.00 (0.98-1.01)	0.98 (0.97-0.99)	0.99 (0.98-1.00)	0.97 (0.95-0.99)
Non-injection drug use*	1.03 (0.85-1.24)	0.90 (0.69-1.17)	1.49 (1.14-1.94)	1.12 (0.86-1.45)	0.92 (0.55-1.53)
Injection drug use*	1.47 (1.21-1.79)	1.07 (0.82-1.39)	1.36 (1.08-1.70)	1.13 (0.89-1.43)	1.78 (1.20-2.64)
**Structural Factors**					
Race					
*Indigenous vs White*	1.04 (0.82-1.31)	1.03 (0.73-1.47)	1.15 (0.86-1.53)	1.27 (0.93-1.75)	2.02 (1.27-3.21)
*Asian, Latinx, Black, Other vs White*	1.37 (1.01-1.86)	1.56 (1.03-2.35)	0.27 (0.17-0.42)	0.45 (0.24-0.82)	0.25 (0.04-1.72)
Im/migrated to Canada	1.21 (0.93-1.58)	1.47 (1.03-2.12)	0.25 (0.16-0.41)	0.54 (0.31-0.92)	0.90 (0.27-3.04)
Sexual minority	1.27 (1.02-1.58)	0.99 (0.73-1.35)	1.38 (1.06-1.81)	1.46 (1.08-1.97)	1.34 (0.88-2.06)
Gender minority	1.27 (0.95-1.72)	1.17 (0.78-1.76)	1.21 (0.86-1.70)	1.35 (0.95-1.91)	1.08 (0.62-1.88)
Diagnosed with any mental health issue	1.10 (0.88-1.37)	1.12 (0.84-1.51)	2.80 (2.08-3.77)	2.10 (1.48-2.98)	2.08 (1.26-3.42)
Unstable housing*	1.16 (0.95-1.42)	0.82 (0.63-1.09)	1.25 (0.97-1.59)	1.04 (0.76-1.42)	2.18 (1.13-4.21)
Experienced violence from aggressor posing as client*	1.62 (1.20-2.19)	1.17 (0.75-1.82)	1.51 (1.06-2.16)	1.23 (0.79-1.90)	2.18 (1.36-3.50)
Any male intimate partner violence*	1.41 (1.02-1.95)	1.54 (1.04-2.28)	1.53 (1.07-2.18)	2.23 (1.58-3.15)	2.36 (1.33-4.21)
Any violence by any perpetrator*	1.40 (1.14-1.72)	1.32 (1.03-1.70)	2.44 (1.93-3.07)	1.85 (1.41-2.43)	3.53 (2.29-5.46)
Primary place of soliciting clients*					
*Indoor (informal or formal) vs street/public*	0.84 (0.64-1.10)	1.02 (0.73-1.43)	0.48 (0.35-0.66)	0.66 (0.45-0.98)	0.67 (0.36-1.25)
*Independent vs street/public*	0.81 (0.66-0.99)	0.86 (0.67-1.11)	0.97 (0.77-1.21)	0.78 (0.61-0.99)	0.56 (0.40-0.80)

* Time updated to last 6 months

1 N = 518, 2N = 460, 3N = 403

NOTE: Non-injection drug use excludes alcohol and cannabis

NOTE: Everyday discrimination OR to be interpreted as for every one-point increase in everyday discrimination score

**Fig 1:**
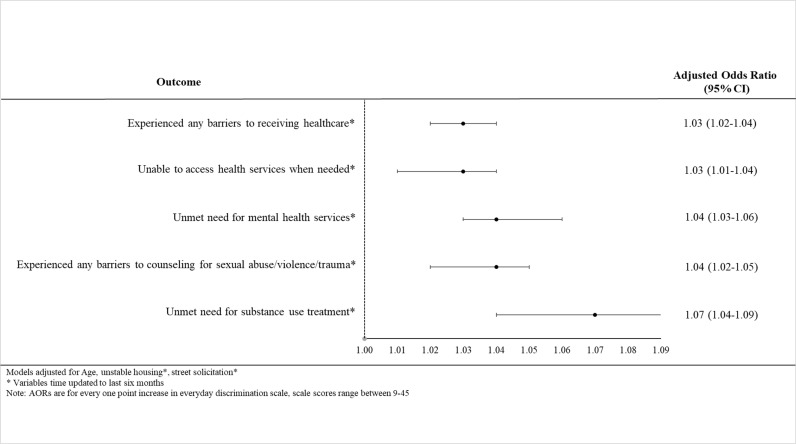
Multivariable GEE models of the association between everyday discrimination and primary care, mental health, and substance use services access among women sex workers in Vancouver, Canada, AESHA, (2015-2024).

In multivariable GEE analyses adjusted for confounders, higher everyday discrimination scores were significantly associated with experiencing poor access to and utilization of primary, mental health, and substance use services (Fig 1). In separate adjusted models, every one-point increase in discrimination score was associated with increased odds of experiencing barriers to health services (adjusted odds ratio (AOR):1.03, 95% CI:1.02-1.04), unable to access health services when needed (AOR:1.03, 95% CI:1.01-1.04), unmet need for mental health services (AOR:1.04, 95% CI:1.03-1.06), experiencing any barriers to counseling for sexual trauma (AOR:1.04, 95% CI:1.02-1.05), and unmet need for substance use treatment (AOR:1.07, 95% CI:1.04-1.09).

## Discussion

In this prospective cohort study of sex workers, higher reported levels of discrimination were associated with reduced access to primary, mental health, and substance use services, and the majority faced barriers to healthcare services. Almost 40% of sex workers experienced discrimination from police, while over half experienced discrimination in healthcare settings. Our findings are consistent with previous research demonstrating that increased discrimination is linked to adverse health outcomes for marginalized populations [8,10,14], including sex workers. Experiencing discrimination was associated with reduced access to primary care, mental health, and substance use services, indicating the importance of multi-level anti-discrimination interventions, including structural changes to decriminalize and destigmatize sex work.

Over this 8.5-year study, 86.9% of sex workers reported any barriers to receiving healthcare and 39.2% reported being unable to access health services when needed. While these findings suggest that sex workers face serious barriers to healthcare, and that discrimination may play a crucial role in this – the finding that 60% of participants self-reported being able to access health services when needed is important. This suggests that many sex workers are successfully overcoming barriers to care, which in many cases may result from ongoing community organizing and outreach supports. Previous studies among marginalized women in Vancouver Canada have found peer-led mobile outreach to play a critical role in accessing drug treatment and supporting HIV antiretroviral therapy adherence [31,32]. Qualitative research from Vancouver has also highlighted the role of community in-reach services in supportive housing, which have been shown to foster destigmatizing care, drop-in hours, and close proximity, enabling participants to engage with health services more consistently [33]. The present study further highlights the importance of interventions that build on these existing community interventions and assets to strengthen accessibility of health services for marginalized people.

Our study builds upon previous sex work discrimination research, which has often focused on HIV-related outcomes [19,20] and is, to our knowledge, the first study among a longitudinal prospective cohort in a high-income country to evaluate the relationship between discrimination and health service access among sex workers, contributing a unique and critical understanding of how discrimination may influence sex workers’ health. The present study reinforces previous literature which has shown associations of discrimination with poor health outcomes such as chronic disease [13] and mental health diagnosis [14]. This research contributes a novel understanding that sex workers who experience everyday discrimination have decreased access to healthcare, building upon previous studies which identified an association between fear of discrimination and lack of healthcare access [19–21]. Our study assessed a range of healthcare services, showing how everyday discrimination not only affects primary healthcare access, but also access to mental health and substance use-related services. Given the well-documented and inequitable burden of HIV, STIs, and mental health issues among sex workers [1,2], understanding and addressing everyday discrimination to improve overall health, mental health, and substance use outcomes globally is vitally important.

We found sex workers reported discrimination for a variety of reasons beyond sex work (i.e., race, sexual minority). This complements previous literature which has found marginalized people often live with multiple stigmatized identities [34]. Future research should more closely examine the role of intersectionality affecting health outcomes among sex workers, ideally using mixed-methods to richly examine the phenomenon [34].

### Limitations and strengths

The reporting of sensitive and stigmatized behaviors is subject to potential information recall bias; the team of community-based and experiential frontline staff are highly skilled in building rapport with participants and being non-stigmatizing, which likely mitigated this to the extent possible. Our measure of discrimination is likely a conservative estimate of discrimination faced by sex workers in this context, as literature has shown that sex workers experience additional violence and discrimination in the workplace, while our measure asked about *everyday* discrimination faced every day in all aspects of life [35]. Given the limited research on this topic, our analysis provides a novel quantitative understanding of the relationship between everyday discrimination and access to healthcare services utilizing 8.5 years of prospective cohort data.

## Public health implications

This study highlights the importance of discrimination as a structural determinant of sex workers’ access to primary care, mental health, and substance use services. Research has suggested that policies criminalizing sex work increase stigma for sex workers and prevent access to occupational health practices [1]. As discrimination represents enacted stigma, interventions addressing public opinion and attitudes towards sex work are needed which target ‘upstream’ factors, including eliminating laws that single out and criminalize sex work, which perpetuate stigma and extensive health-related harms [26,27]. Interventions that perpetuate existing myths and stereotypes that conflate sex work with trafficking, conceptualize all sex workers categorically as ‘victims’, or which characterize sex work as inherently immoral must be challenged through continued organization of sex workers and legislative changes that address sex work as work, empower sex workers to control their own narratives, and amplify their voices. Sex work decriminalization has been recommended as a ‘best practice’ by various global health bodies, and the United Nations Programme on HIV/AIDS (UNAIDS) action plan to eliminate discrimination emphasizes that discrimination occurs in settings including the criminal legal system and must be addressed at such systemic levels [36]. We also recommend widespread implementation of anti-discrimination protections suggested by the Australian Sex Workers Association. These guidelines, which are most easily implemented in places with full decriminalization of sex work, include protection of sex workers from sexual harassment, improved access to filing complaints (including representation in the complaint process), privacy protections, and protections for families and associates [37].

Additionally, the UN, World Health Organization, and Global Network of Sex Work Projects recommend health services based on non-discrimination and the right to health. To achieve this, anti-discrimination, culturally appropriate, and sex worker-led provider trainings are needed. Given the high proportion of sex workers who experience discrimination from police and in healthcare, we recommend specific trainings targeting these institutions, as has recently been rolled out by the Vancouver Sex Work Community Alliance with healthcare providers [38]. To ensure non-discriminatory health service delivery, these systems should be monitored and provide effective and accessible complaint procedures for marginalized populations including sex workers.

In this prospective cohort study of sex workers in Vancouver, Canada, everyday discrimination was associated with decreased access to and unmet need for various health services, including primary care and barriers to mental health support and substance use treatment. These findings underscore the importance of addressing discrimination as a social-structural determinant of health to improve the health outcomes of marginalized populations that bear an increased burden of discrimination. To improve the health of sex workers as a population and strengthen sexual health globally, multi-level anti-discrimination interventions are needed, including trainings for health service providers and high-level policy changes to decriminalize sex work.

## Supporting information

S1 Checklist Inclusivity in global research checklist. (PDF)

S1 Table Everyday discrimination scale, adapted from Williams and Colleagues (1997) for AESHA with period prevalence of each scale subcategory, (2015–2024). (DOCX)

## Footnotes

1 Everyday discrimination scale was not asked at baseline, but at every follow-up

2 Given the low proportion of participants who identiﬁed as Black in our sample (consistent with the Black population of BC (<2%)), we combined Black and Women of Color in analysis due to confidentiality and statistical power considerations.

3 Response added based on community feedback to describe the response of other people projecting their own issues onto sex workers
