# Supplementary material for: Everyday discrimination and barriers to primary care, mental health, and substance use services: Findings from a community-based cohort of sex workers in Vancouver, Canada (2015–2024)
Source: PLOS Glob Public Health. 2025 Jun 16;5(6):e0004647. doi: 10.1371/journal.pgph.0004647 (PMC12169575; doi:10.1371/journal.pgph.0004647)
Supplement: S1 Table — (DOCX) [file pgph.0004647.s002.docx]

**S1 Table. Everyday Discrimination Scale, Adapted from Williams and Colleagues (1997) for AESHA with Period Prevalence of each Scale Subcategory, (2015-2024)**

| **‘**In the last 6 months, in your day-to-day life how often do any of the following things happen to you?’ | **Ever (%)** |
| --- | --- |
| People call you names | 331 (65.3) |
| People threaten or harass you | 322 (63.4) |
| You receive poorer service from people | 372 (73.2) |
| People do not treat you with respect | 389 (76.4) |
| People act like you are dishonest | 355 (69.9) |
| People act like they are afraid of you | 322 (63.4) |
| People are not polite to you | 388 (76.4) |
| People act like they are better than you | 392 (77.2) |
| People act like you are not as smart as they are | 376 (74.0) |

*Ever column created by combining occasionally, sometimes, usually (at least once a week), and always (almost every day)

**Each subcategory has a different denominator N, as missing were not included
